# Molecular Prevalence and Identification of *Ehrlichia canis* and *Anaplasma platys* from Dogs in Nay Pyi Taw Area, Myanmar

**DOI:** 10.1155/2021/8827206

**Published:** 2021-02-08

**Authors:** Myint Myint Hmoon, Lat Lat Htun, May June Thu, Hla Myet Chel, Yu Nandi Thaw, Shwe Yee Win, Nyein Chan Soe, Yadanar Khaing, Su Su Thein, Saw Bawm

**Affiliations:** ^1^Department of Pharmacology and Parasitology, University of Veterinary Science, Nay Pyi Taw 15013, Myanmar; ^2^Department of International Relations and Information Technology, University of Veterinary Science, Nay Pyi Taw 15013, Myanmar

## Abstract

Ticks are vectors of different types of viruses, protozoans, and other microorganisms, which include Gram-negative prokaryotes of the genera *Rickettsiales*, *Ehrlichia*, *Anaplasma*, and *Borrelia*. Canine monocytic ehrlichiosis caused by *Ehrlichia canis* and canine cyclic thrombocytopenia caused by *Anaplasma platys* are of veterinary importance worldwide. In Myanmar, there is limited information concerning tick-borne pathogens, *Ehrlichia* and *Anaplasma* spp., as well as genetic characterization of these species. We performed nested PCR for the *gltA* gene of the genus *Ehrlichia* spp. and the 16S rRNA gene of the genus *Anaplasma* spp. with blood samples from 400 apparently healthy dogs in Nay Pyi Taw area. These amplicon sequences were compared with other sequences from GenBank. Among the 400 blood samples from dogs, 3 (0.75%) were positive for *E. canis* and 1 (0.25%) was positive for *A. platys*. The partial sequences of the *E. canis gltA* and *A. platys* 16SrRNA genes obtained were highly similar to *E. canis* and *A. platys* isolated from different other countries.

## 1. Introduction

Tick-borne bacteria and parasites are important pathogens of domestic dogs and are potentially of public health significance. At least five bacterial species, *Ehrlichia canis*, *E. chaffeensis*, *E. ewingii*, *Anaplasma platys*, and *A. phagocytophilum*, have been reported in domestic dogs [1]. *E. canis* is transstadially transmitted by the brown dog tick, *Rhipicephalus sanguineus*, and all feeding stages of tick can transmit the infection to susceptible dogs, and nymphal and adults can transmit *E. canis* for at least 155 days after detachment from an infected host [2]. *Ehrlichia canis* was the first *Rickettsiales* described in dogs and is the causal agent of canine monocytic ehrlichiosis (CME), which has a worldwide distribution, particularly in tropical and subtropical regions [3–5]. These bacteria are classified in the family Anaplasmataceae, which includes obligate intracellular prokaryotic parasites that reside within a parasitophorous vacuole [6]. In canine hosts, *E. canis* is infective for monocytes [7].

*Anaplasma platys* infections in dogs are distributed throughout the world. *A. platys* is the causative agent of canine infectious cyclic thrombocytopenia, which infects the platelets, but infected dogs showed no clinical signs [8]. *A. platys* infection is difficult to detect not only “in vivo” because of the low bacteremias but also serologically because of cross-reaction with other *Anaplasma* species [9, 10]. Thus, a PCR assay is a reliable method for the detection of *A. platys* infection in dogs [11].

The objectives of this study were to determine the presence of *E. canis* and *A. platys* in dogs and to compare Myanmar isolates with those from other regions. Herein, we used nested PCR and phylogenetic analysis to detect the molecular characteristics of *E. canis* and *A. platys* infections from dogs in the Nay Pyi Taw area, Myanmar.

## 2. Materials and Methods

### 2.1. Study Site and Sample Collection

**Figure 1:**
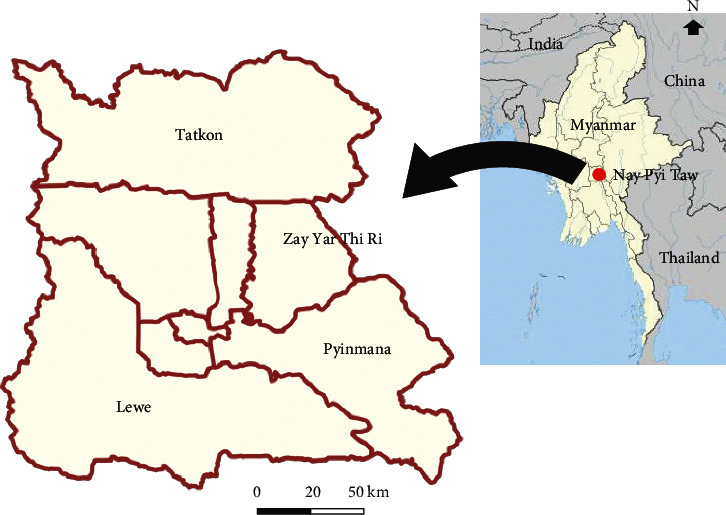
Map of the sampling area.

This study was conducted in four townships: Lewe (19.6349°N, 96.1076°E), Pyinmana (19.7414°N, 96.2004°E), Tatkon (20.1284°N, 96.1527°E), and Zay Yar Thi Ri (19.62°N, 96.02°E) (Figure 1). Between December 2016 and March 2017, blood samples were collected from 400 apparently healthy dogs. From the urban and rural areas of each township, 100 dogs were sampled. Most of the dogs are free roaming in rural Myanmar, while they belong to someone. Before taking blood samples, we explained our aim of the study to the owner, and we have already obtained consent for the experiment from the dog owners. Blood collection (approximately 3 ml) was performed from the sphenoid vein and jugular vein and put into ethylene diamine tetraacetic acid (EDTA) tubes. All collected samples were transferred to the laboratory at 4°C. Within 24 hr of sample collection, DNA extraction was conducted. During blood collection, dogs were examined for the presence of ticks, and if present, ticks were collected in plastic containers containing a small piece of wet sponge for further taxonomic identification.

### 2.2. DNA Extraction from Canine Blood

Extraction of DNA from the blood samples was conducted by using a commercial DNA extraction reagent (DNAzol®) (Molecular Research Center, Inc., USA) according to the manufacturer's instructions [12]. The volume of blood used for DNA extraction was 100 *μ*l. The extracted DNAs were eluted in 200 *μ*l elution buffer and stored at −80°C. DNA concentration was estimated using a NanoDrop 2000 spectrophotometer (ThermoFisher Scientific, MA, USA).

### 2.3. Polymerase Chain Reaction (PCR) to Amplify *Ehrlichia* and *Anaplasma* spp

For *Ehrlichia* spp., seminested PCR amplification of the *gltA* gene fragment was performed by using a SimpliAmp Thermal cycler (Applied Biosystem, USA) as previously described [13]. Outer primers, EHRCS-131F (CAGGATTTATGTCTACTGCTGCTTG) and EHRCS-1226R (CCAGTATATAAYTGACGWGGACG), were used for the amplification of the first-round product (1,096bp), and inner primers, EHRCS-131F (CAGGATTTATGTCTACTGCTGCT TG) and EHRCS-879R (TIGCKCCACCATGAGCTG), were used for the amplification of the second-round product (748bp). For *Anaplasma* spp., seminested PCR amplification of the 16S rRNA gene fragment was performed according to Inokuma et al. [14]. Outer primers, fD1 (AGAGTTTGATCCTGGCTC AG) and EHR16SR (TAGCACTCATCGTTTA CAGC), were used for the first-round product (1,000bp), and inner primers, EHR16SD (GGTACC(C/T)ACAGAAGAAGTCC) and Rp2 (ACGGCTACCTTGTTACGACTT), were used for the second-round product (1,000bp). PCR mixture contained approximately 20–100 ng of extracted DNA, 0.3 *μ*M of each primer, 0.025 U/*μ*L of Tks Gflex™ DNA polymerase (Takara Bio Inc., Tokyo, Japan), and 1 × Gflex buffer in a volume of 25 *μ*L. For both species, cycling conditions were denaturation for 1 min at 94°C, followed by 98°C for 10 s. The annealing temperature used was 50°C for 15s for *Ehrlichia* spp. and 55°C for *Anaplasma* spp., followed by 68°C for 90s for 40 cycles, and a final extension for 5 min at 68°C. The PCR products were visualized by electrophoresis on 1.5% agarose gels stained with RedSafe (NIPPON Genetics, Duren, Germany).

### 2.4. Sequencing and Phylogenetic Analysis

Positive PCR products were purified using the NucleoSpin® and PCR Clean-up Kit (MACHEREY-NAGEL, Duren, Germany) according to the manufacturer's instructions. Purified PCR products were sequenced with the ABI 3130 genetic analyzer (Model 3130; Applied Biosystems, Foster City, CA, USA) with forward and reverse primers. Nucleotide sequences were compared with GenBank entries using NCBI BLAST (http://www.ncbi.nlm.nih.gov/blast). Phylogenetic analysis is performed using the DNA sequence.

Multiple sequence alignments of positive amplicons and *gltA* and 16S rRNA sequences from GenBank were performed using the ClustalW Version 1.8 [15]. Phylogenetic trees were inferred using neighbor-joining (NJ) analysis using MEGA software version 7.0 [16]. The distance matrix of nucleotide divergences was calculated according to Kimura's two-parameter model furnished by MEGA. A bootstrap resampling technique of 1000 replications was performed to statistically support the reliabilities of the nodes on the trees.

## 3. Ethical Considerations

Experiments were carried out in accordance with the guidelines laid down by the Institutional Ethics Committee. All studies using animal subjects were approved by the Ethics Committee of University of Veterinary Science, Nay Pyi Taw, Myanmar (approval no. 309/Katha (postgraduate)/2016.

## 4. Results and Discussion

**Figure 2:**
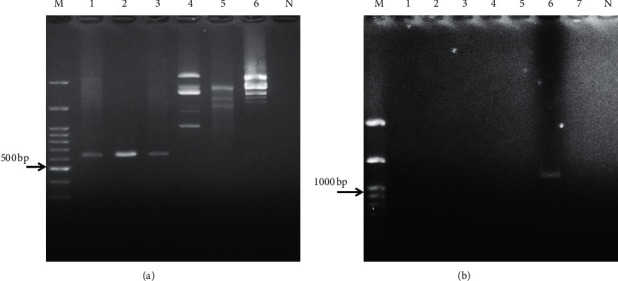
Gel electrophoresis results for nested PCR of *E. canis* (a): lanes 1–3 = positive samples, lanes 4–6 = negative samples, and *N* = negative control, and *A. platys* (b): lane 6 = positive, lanes 1–5, and 7 = negative samples, and *N* = negative control; *M* = 100bp marker.

Of the 400 dogs analyzed, 3 samples (0.75%) were positive for *E. canis*, while 1 sample (0.25%) was positive for *A. platys* (Table 1, Figures 2(a) and 2(b)). Descriptive data of sampled dogs and tick infestation are shown in Table 2. All PCR-positive samples for *A. platys* and *E. canis* were confirmed by sequencing results.

**Figure 3:**
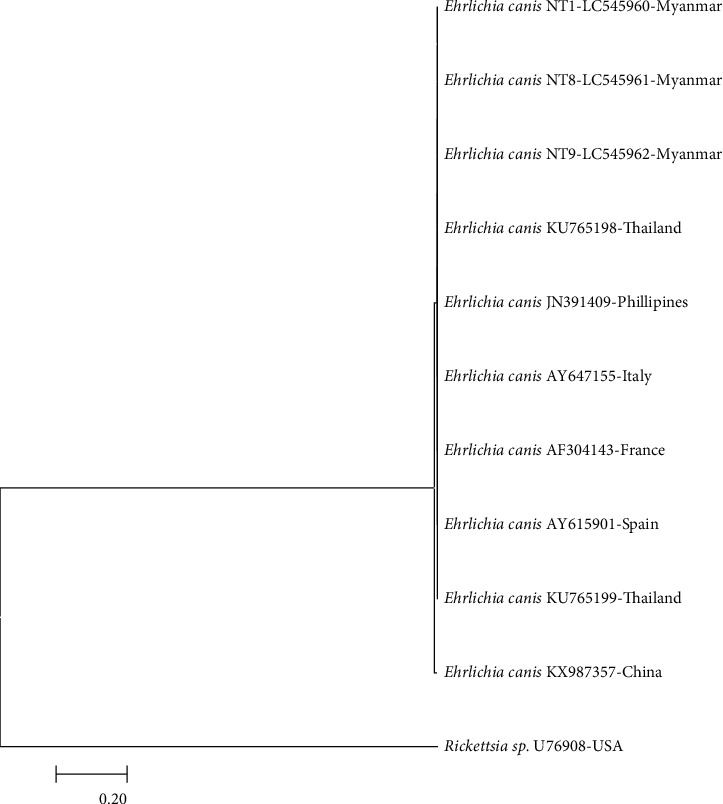
Phylogenetic tree based on the *Ehrlichia gltA* sequence. Sequences from the *Ehrlichia* genera were compared with the neighbor-joining method with distance matrix calculation by Kumar-two parameters, operated by MEGA software (Version 7), using *Rickettsia* sp. as the outgroup. Scale bar indicates the number of mutations per sequence position. The numbers at the nodes represent the percentage of 1000 bootstrap resamplings.

**Figure 4:**
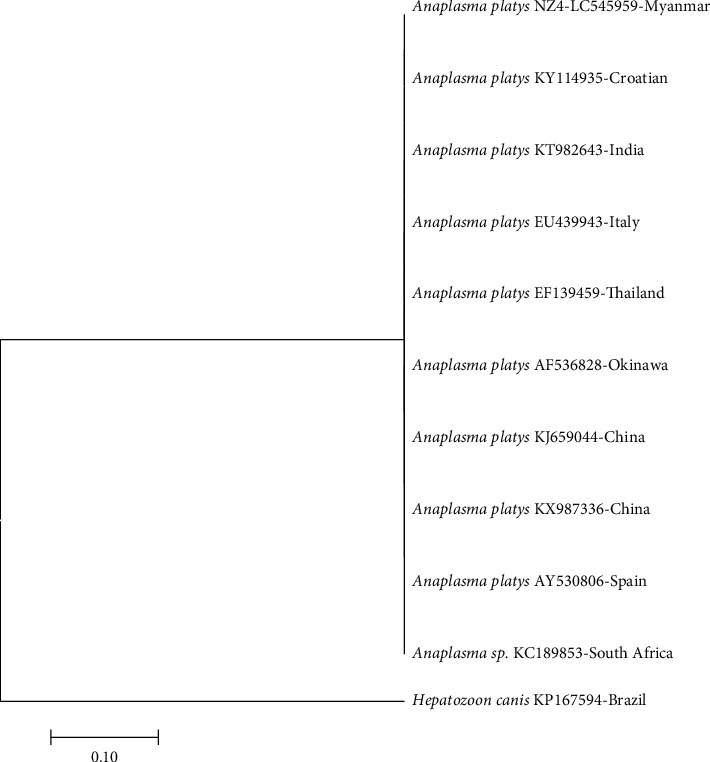
Phylogenetic tree based on the *Anaplasma* 16S rRNA sequence. Sequences from the *Anaplasma* genera were compared with the neighbor-joining method with distance matrix calculation by Kumar-two parameters, operated by MEGA software (version 7), using *Hepatozoon canis* as the outgroup. Scale bar indicates the number of mutations per sequence position. The numbers at the nodes represent the percentage of 1000 bootstrap resamplings.

In the phylogenetic trees based on *gltA* genes, *E. canis* was detected in dogs T1, T8, and T9 grouped in the same cluster as other *E. canis* strains, supported with a 100% bootstrap value. The *A. platys* 16SrRNA gene from dog Z4 was found in the same cluster as other *A. platys* strains, supported with a 100% bootstrap value. The sequences obtained were similar to those of *E. canis* strains from Philippines, Italy, Spain, France, China, and Thailand (GenBank accession no. JN391409, AY647155, AY615901, AF304143, KX987357, KU765198, and KU765199) with similarities of 98.46–100% (Figure 3). *A. platys* 16S rRNA sequences obtained were similar to those of *A. platys* strains from India, Thailand, Italy, Okinawa, Croatia, China, Spain, and South Africa (GenBank accession no. KT982643, EF139459, EU439943, AF536828, KY114935, KJ659044, KX987336, AY530806, and KC189853) with similarities of 99.68–100% (Figure 4). The results from phylogenetic analysis confirmed that the amplified genes belong to the respective species. Sequences generated in the present study have been submitted to GenBank under accession numbers LC545959 to LC545962.

In this study, molecular identification from 400 local dog samples demonstrated a prevalence of 0.75% for *E. canis* infection and 0.25% for *A. platys* infection. There was no mixed infection in this study. According to the findings of this study, *E. canis* was found as more common canine tick-borne pathogen when compared to *Anaplasma* spp. In this study, the present results indicate a low prevalence of subclinical infection in dogs. In Turkey, the prevalence of *E. canis* from asymptomatic dogs was 4.9%, *A. platys* was 0.5%, and mixed infections of *E. canis* and *A. platys* were detected as 0.3% [17]. In Brazil, only 4.8% of the dogs were seroreactive to *E. canis* [18]. Previous studies have described that the molecular prevalence of *E. canis* ranged from 3.1% to 88% [19–23]. The variation might be due to the sample size, climatic conditions that directly influence the tick population, and the time of sample collection.

A higher prevalence of *E. canis* and *A. platys* was also reported by some workers. In Praia, Austria, Gotsch et al. [24] indicated that the PCR examination for *E. canis* in dogs was 26.2% and *A. platys* was 7.7%. In North Carolina, USA, 33% of 27 dogs was *A. platys* PCR-positive [25]. In Okinawa, Japan, 32% of 200 stray dogs was positive by *A. platys*-specific PCR [26]. In fact, in the previous studies, the positive dogs were sick animals with clinical signs compatible with vector-borne diseases and admitted for medical treatment, while in the present study, all the dogs sampled were apparently healthy.

*Anaplasma platys* is a thrombocytotrophic bacteria of dogs that is characterized by clinical abnormalities such as fever, anorexia, petechial haemorrhages, and uveitis [27]. The detection of *A. platys* infection in dogs was the first time in Myanmar. In this study, it was lower prevalence of *A. platys* (0.25%) than that in Italy (4%), Nigeria (6.6%), and Venezuela (16%). In Portugal, *A. platys* DNA has been detected in clinically suspected dogs living in the north and south of Portugal [28], while the overall national seroprevalence of *Anaplasma* spp. has ranged from 4.5% in apparently healthy to 9.2% in clinically suspect dogs [29]. The lower prevalence of *A. platys* in this study might be due to different DNA extraction methods, and the local breed of the examined dogs in this study seemed to be genetically resistant to tick-borne pathogens. In this study, the older dogs were more likely to be positive and could have a greater risk of tick-borne diseases. Moreover, younger dogs might be maternally immune to tick infection. Since local dogs are free roaming in rural areas, they have never been treated or removed of ticks, and they may naturally be resistant to tick-borne diseases. However, further studies are necessary to identify the infections of *E. canis* and *A. platys* from both ticks and hosts.

In this study, all the tick samples collected during sampling were morphologically and molecularly diagnosed *R. sanguineus* (data not shown). However, the occurrence of tick infestation in dogs in the study area was low (11%, 44/400) [30]. A total of 237 ticks were collected from 44 dogs with an average of 4-5 ticks per dog. Three out of four positive dogs were infected with ticks in the studied areas. These data suggest that *E. canis* and *A. platys* might be shared by the same vector, *R. sanguineus*. In Myanmar, Chel [31] studied that the prevalence of *R. sanguineus* tick in Nay Pyi Taw area was 0% in the summer season, 84.7% in the rainy season, and 15.3% in the winter season. Asebe et al. [32] also discussed that in tropical climates, there is a marked decrease in tick population at the end of the rainy season and with progressive fall to almost zero in the dry season. In fact, as stated by Huang et al. [33], one of the reasons for the low prevalence of *E. canis* and *A. platys* might also be due to a very small number of *R. sanguineus* ticks collected in the present study. Moreover, this might be due to climatic conditions during the sampling period (from December to March), which were not favourable for development and survival of *R. sanguineus*.

The partial sequences of the *gltA* and 16SrRNA genes obtained in this study were highly similar to strains of *E. canis* and *A. platys* isolated from different other countries. This implied that the *E. canis* and *A. platys* isolates found in Myanmar were not divergent from the strains of other countries. This might be due to the fact that transboundary movement of domestic and wild animals might carry infected ticks between Myanmar and neighboring countries. The vectors might distribute genetically similar pathogens among these countries.

## 5. Conclusion

The findings of this study are basic information regarding *E. canis* and *A. platys* infection in Myanmar. Moreover, further research related to the genetic diversity of *E. canis* and *A. platys* from another area of Myanmar should be conducted.

## Figures and Tables

**Table 1:** Prevalence of *E. canis* and *A. platys* in four townships within Nay Pyi Taw area.

Location	No. of collected samples	No. (%) of positive samples	
		*E. canis*	*A. platys*
Pyinmana	100	0 (0)	0 (0)
Zay Yar Thi Ri	100	0 (0)	1 (1)
Lewe	100	0 (0)	0 (0)
Tatkon	100	3 (3)	0 (0)
Total	400	3 (0.75)	1 (0.25)

**Table 2:** Description of sampled dogs and tick infestation.

No. of examined dogs	Age		Sex		Breed		Tick infestation	
	>1 year	<1 year	Male	Female	Local	Exotic	Yes	No
400	251	149	220	180	400	0	44	356

## Data Availability

Sequences generated in the present study have been submitted to GenBank under accession numbers LC545959 to LC545962.
